# Longitudinal associations of concurrent falls and fear of falling with functional limitations differ by living alone or not

**DOI:** 10.3389/fpubh.2023.1007563

**Published:** 2023-04-12

**Authors:** Kehan Liu, Wenting Peng, Song Ge, Chunxiao Li, Yu Zheng, Xiaoting Huang, Minhui Liu

**Affiliations:** ^1^Xiangya School of Nursing, Central South University, Changsha, China; ^2^Department of Natural Sciences, University of Houston-Downtown, Houston, TX, United States

**Keywords:** falls, fear of falling, functional limitations, living alone, older adults

## Abstract

**Background:**

Falls and fear of falling (FOF) are independent risk factors for functional limitations in older adults. However, the combined effect of falls and FOF on functional limitations and the moderating role of living alone or not is unclear. We aimed to examine (1) the independent and combined effect of falls and FOF on functional limitations in older adults and (2) whether living alone moderates these associations.

**Methods:**

We used data from the National Health and Aging Trends Study (NHATS) and included 5,950 U.S. community-dwelling older adults aged 65 and older from Round 1 (Year 2011) and Round 2 (Year 2012). Falls and FOF were ascertained by asking participants whether they had any falls in the last year and whether they had worried about falling in the previous month at R1. Assessed functional limitations included any difficulties with mobility, self-care, or household activities at R2. Poisson regression models were used to examine the longitudinal associations of falls and FOF with functional limitations and the moderation effects of baseline living alone.

**Results:**

Of the 5,950 participants, 16.3% had falls only; 14.3% had FOF only; 14.3% had both, and 55.1% had neither at baseline. In the adjusted model, those who experienced concurrent falls and FOF in R1 had a higher risk of functional limitations at R2 than those with neither (Mobility: Incidence risk ratio [IRR] = 1.34, 95% CI: 1.24–1.45; Self-care: IRR = 1.18, 95% CI: 1.11–1.26; Household: IRR = 1.20, 95% CI: 1.11–1.30). Moreover, living alone significantly moderated the longitudinal associations of concurrent falls and FOF with mobility activity limitations.

**Conclusion:**

The findings suggest that strategies to improve falls and FOF together could potentially help prevent functional limitations. Older adults who live with others and have falls or FOF should receive interventions to promote their mobility activities.

## 1. Introduction

Functional limitations are defined as reduced ability to perform basic daily activities required to live independently in a community (1). Approximately 25.7% of US adults have functional limitations (e.g., mobility, self-care), and more than half of them are 65 years and older (2). Functional limitations are associated with increased risk of stress, disability, depression, and mortality in older adults (3). In addition, the annual healthcare expenditure related to functional limitations in older adult accounts for 46.3% of the total U.S. healthcare expenditure (4). Healthcare expenditure is higher for older adults with functional limitations than those without (4, 5). Therefore, it is important to identify the modifiable risk factors for functional limitations in older adults and intervene in these risk factors.

Falls have many negative health effects, including deteriorating functional limitations in older adults (6). In the US, about 1.8 million older adults visit emergency departments for nonfatal fall injuries every year (7). Over 40% of them reported having functional limitations two months after the fall (8). Fear of falling (FOF) refers to the unhealthy avoidance of activities due to fear of falling (9). Findings of the International Mobility in Aging Study (*n* = 1,601) suggested that FOF is positively associated with the risk of functional limitations. Older adults with FOF excessively restricted their activities over time (10). In a cohort of 864 community-dwelling older adults in the US, our previous study findings showed that FOF independently predicted functional limitations after adjusting for falls and other covariates; and falls independently predicted functional limitations after adjusting for FOF and other covariates (11). Falls or FOF have been identified as modifiable risk factors for functional limitations (12, 13). Increasing evidence has characterized a bidirectional link between falls and FOF (14, 15). Specifically, falls in the previous year are a predictor of FOF and FOF is a predictor of subsequent falls (16). Falls and FOF often co-occur and are related, and the development of either may trigger a cascading effect that may increase risk of functional limitations (17). Considering the complicated association between falls and FOF, it is important to figure out their independent and combined effects on functional limitations to improve disability interventions for maximal impact. However, previous studies only separately investigated the influence of falls or FOF on functional limitations (8, 10, 11, 18, 19), whether falls and FOF combinedly predict functional limitations remains unclear.

Living alone in later life is often seen as an undesirable state, as most older adults who live alone are at a higher risk of falls and FOF (20–22). Studies have found that older adults who lived alone were 2–2.25 times more likely to fall and even experience multiple falls (20). A cross-sectional study of over 4,000 older adults demonstrated that those who lived alone (62.2%) had more FOF than those who did not live alone (23). Nevertheless, living alone is not an absolutely negative factor to health (24). There is evidence that people who lived alone 10 years ago were just as healthy as those who lived with others (25). Indeed, some studies showed that older adults who lived alone maintained functional independence and were less likely to experience functional limitations than those who did not live alone (26, 27). They monitored their health more diligently, were more mentally determined, and actively trained themselves to prevent functional limitations (28). Based on the above evidence, living alone might predict falls and FOF but alleviate the risk of functional limitations in older adults. To the best of our knowledge, the moderating effects of living alone in the association of falls and FOF on functional limitations have not been examined.

To address these key evidence gaps, we aimed to examine (1) whether falls and FOF in the previous year independently and combinedly predict functional limitations in the following year in older adults; and (2) whether living alone moderates the associations of falls and FOF with functional limitations. We hypothesized that falls and FOF independently and combinedly predict future functional limitations and living alone moderates these relationships.

## 2. Methods

The National Health and Aging Trends Study (NHATS) is an ongoing longitudinal study of Medicare beneficiaries aged 65 and older in the United States (29). The first round started in 2011 and aimed to investigate the disability trends of older adults in late life. We used the data from Round one (2011) and Round two (2012) of NHATS for this study. Of the 8,245 participants in Round one, 7,609 lived in the community and completed the sample person interview. Their response rate in Round two was 80.3% (*n* = 6,113). Then, 91 participants residing in nursing homes in Round two were excluded; 6,022 participants were eligible for further analysis. A total of 5,950 participants were finally included in the analysis (5,950 of 6,022; 98.8%) after excluding those with missing values on the functional limitations at follow-up (31 of 6,022; 0.5%) or independent variable (falls and FOF) and moderator (living alone) at baseline (41 of 6,022; 0.7%). Compared to those included in this analysis, the excluded participants were more likely to be older, female, and less educated (all *P* < 0.05). The NHATS study was approved by the Johns Hopkins University Institutional Review Board. The current study used publicly available and de-identified data and was deemed exempt by Xiangya School of Nursing Central South University.

###  2.1. Measures

#### 2.1.1. Dependent variable: Functional limitations

Functional limitations were assessed by limitations in three mobility activities (going outside, getting around inside, and getting out of bed), four self-care activities (eating, dressing, toileting, and bathing), and five household activities (laundering, shopping, cooking, banking, and taking medications). Each activity was assessed by asking participants whether they performed any activities with any difficulty, whether they needed help from others, and whether they used any assistance devices over the last month. For all activities except getting out of bed, toileting, and eating, participants were also asked if they did them less frequently than a year ago.

Consistent with previous studies (11, 30–32), a four-category hierarchal scale was used to define the level of each activity. The score of each activity ranged from zero to three. A score of zero represented no limitations, indicating that participants could perform the activity with no difficulty, help, assistance devices, or reduction in frequency. A score of one represented successful accommodation, indicating that participants could perform the activity less frequently or with assistance devices but with no difficulty or help. A score of two represented difficulty meaning that participants had difficulty performing the activity but did not receive assistance. A score of three represented assistance, indicating that participants performed the activity with others' help or did not perform the activity. Therefore, the mobility score (with three questions) ranged from zero to nine. The self-care score (with four questions) ranged from zero to 12. The household score (with five questions) ranged from zero to 15. A higher score indicated more functional limitations.

#### 2.1.2. Independent variables: Falls and FOF

Falls were measured by the question-“have you fallen down over the last 12 months?” FOF was measured by the question- “did you worry about falling down in the last month?” Based on their response, the participants were classified into four categories-neither (neither falls nor FOF), falls only (had falls but not FOF), FOF only (had FOF but not falls), and both (had both falls and FOF).

#### 2.1.3. Covariates

Demographic covariates included age (65–79 or over 80), sex (female or male), race/ethnicity (White, Black, Hispanic, or others), education (less than high school, high school, or higher than high school), and living alone (yes/no). Health-related covariates included obesity [body mass index (BMI) ≥30 kg/m^2^, yes or no], depressive symptoms (yes or no), anxiety (yes or no), pain (yes or no), visual impairment (yes or no), hospitalization in the last 12 months (yes or no), dementia status (yes or no), number of chronic diseases (no diseases, 1–3 diseases, or ≥4 diseases) and Short Physical Performance Battery (SPPB).

Living alone was assessed by current living arrangement. Those who were not living with spouse/partner/others were regarded as living alone. Depressive symptoms were measured by the Patient Health Questionnaire-2 (33) and a score of 3 or higher indicated depressive symptoms. Anxiety was measured by the Generalized Anxiety Disorder-2 (33) and a score of 3 or higher indicated anxiety. Pain was measured by the question, “whether you have been bothered by pain in the last month?” Visual impairment was determined by the question, “whether you were blind or unable to see well enough to recognize people across the street or read newspaper print?” Dementia status was assessed by participants' self-reported medical diagnosis of dementia or Alzheimer's disease. Number of chronic diseases was estimated from the total count of chronic diseases, including arthritis, cancer, diabetes, heart attack, heart disease, hypertension, lung disease, osteoporosis, and stroke. SPPB consisted of a balance stand test (hold side-by-side, semi-tandem, or full tandem stances for 10 seconds), a walking speed test (walk 3 m at normal speed for two trials), and a repeated chair stand test (repeat the sit-to-stand five times as fast as possible with arms folded across the chests). The score of each test ranged from 0 (worst) to 4 (best). The score of SPPB ranged from 0 to twelve, with a higher score indicating better physical performance in the lower extremities (17, 34).

### 2.2. Statistical analysis

Frequencies and percentages were used to describe participants' baseline demographic and health information. Chi-squared tests were used to compare the demographic and health-related differences among the four groups (neither, falls only, FOF only, and both). Three Poisson regression models were constructed to examine whether falls and FOF (independent variable) independently and combinedly predict the three outcomes (mobility, self-care, and household limitations). An interaction term between living alone and falls and FOF was then entered into the three models to test the moderating effect. Additionally, stratified analyses were performed to determine the differential magnitude of the relationships between falls and FOF on functional limitations. All models accounted for the sociodemographic factors, health-related factors and outcome of interest at baseline.

Both incidence rate ratio (IRR) and 95% confidence intervals (CI) were reported. To account for missing data, we performed multiple imputation by chained equations (35). The IRR from ten imputed data sets was combined based on Rubin's rule. In our study, all the Poisson regressions were examined using imputed data. A *P* < 0.05 indicated statistical significance. All analyses were conducted using STATA SE version 15.0 (College Station, TX: StataCorp LP).

## 3. Results

### 3.1. Participants' characteristics

Table 1 presented the demographic and health information of the participants. Most participants were 65–79 years old (60.7%), female (58.2%), white (69.0%), and completed higher than high school education (46.8%). Approximately 16.3% of them reported falls only; 14.3% reported FOF only; 14.3% reported both; 55.1% reported neither. Compared to neither, falls only, FOF only, and both were older, less educated, more obese, more depressed, more anxious, more likely to be female and white, and more likely to have pain, visual impairment, hospitalization, dementia, chronic diseases and lower SPPB scores (*P* < 0.001). In terms of living alone, compared to neither, FOF only and both were less likely to live alone (69.1% [neither], 61.7% [FOF only], and 61.5% [Both]). Falls only and neither have similar percentages of older adults not living alone (69.1% [neither] versa 69.8% [falls only]).

**Table 1:** Baseline sample characteristics by baseline falls and FOF status, *n* (%).

**Characteristics**	**Overall, *n =* 5,950 (100.0)**	**Neither, *n =* 3,276 (55.1)**	**Falls only, *n =* 970 (16.3)**	**FOF only, *n =* 852 (14.3)**	**Both, *n =* 852 (14.3)**	***P*-value**
**Age**						
65–79 years	3,611 (60.7)	2,179 (66.5)	590 (60.8)	431 (50.6)	411 (48.2)	< 0.001
80+ years	2,339 (39.3)	1,097 (33.5)	380 (39.2)	421 (49.4)	441 (51.8)	
**Sex**, ***n*** **(%)**						
Female	3,461 (58.2)	1,736 (53.0)	557 (57.4)	599 (70.3)	569 (66.8)	< 0.001
Male	2,489 (41.8)	1,540 (47.0)	413 (42.6)	253 (29.7)	283 (33.2)	
**Race/ethnicity**						
White	4,107 (69.0)	2,203 (67.3)	708 (73.0)	594 (69.7)	602 (70.7)	< 0.001
Black	1,284 (21.6)	770 (23.5)	191 (19.7)	160 (18.8)	163 (19.1)	
Hispanic	346 (5.8)	170 (5.2)	43 (4.4)	66 (7.6)	67 (7.9)	
Other	213 (3.6)	133 (4.1)	28 (2.9)	32 (3.8)	20 (2.4)	
**Education**						
Less than high school	1,538 (26.1)	790 (24.3)	256 (26.6)	224 (26.5)	268 (31.7)	< 0.001
High school	1,600 (27.1)	874 (26.9)	251 (26.1)	256 (30.3)	219 (25.9)	
Higher than high school	2,762 (46.8)	1,583 (48.8)	456 (47.4)	364 (43.1)	359 (42.4)	
**Living alone**						
No	3,992 (67.1)	2,265 (69.1)	667 (69.8)	526 (61.7)	524 (61.5)	< 0.001
Yes	1,958 (32.9)	1,011 (30.9)	293 (30.2)	326 (38.3)	328 (38.5)	
**Obesity**						
No (< 30 kg/m^2^)	4,177 (72.5)	2,385 (75.0)	699 (73.9)	548 (66.4)	545 (67.3)	< 0.001
Yes (≥30 kg/m^2^)	1,586 (27.5)	797 (25.0)	247 (26.1)	277 (33.6)	265 (32.7)	
**Depressive symptom**						
No	5,032 (85.2)	2,944 (90.4)	822 (85.2)	684 (80.9)	582 (69.0)	< 0.001
Yes	877 (14.8)	312 (9.6)	143 (14.8)	161 (19.1)	262 (31.0)	
**Anxiety symptom**						
No	5,183 (87.4)	3,046 (93.3)	868 (89.9)	684 (80.5)	585 (69.0)	< 0.001
Yes	745 (12.6)	218 (6.7)	98 (10.1)	166 (19.5)	263 (31.0)	
**Pain**						
No	2,734 (46.0)	1,851 (56.5)	392 (40.4)	274 (31.2)	217 (25.5)	< 0.001
Yes	3,214 (54.0)	1,423 (43.5)	578 (59.6)	578 (67.8)	635 (74.5)	
**Visual impairment**						
No	5,339 (90.1)	3,049 (93.4)	870 (89.9)	753 (88.6)	667 (78.9)	< 0.001
Yes	589 (9.9)	216 (6.6)	98 (10.1)	97 (11.4)	178 (21.1)	
**Hospitalization**						
No	4,601 (77.4)	2,694 (82.3)	715 (73.8)	651 (76.4)	541 (63.7)	< 0.001
Yes	1,343 (22.6)	579 (17.7)	254 (26.2)	201 (23.6)	309 (36.4)	
**Dementia**						
No	5,664 (95.2)	3,180 (97.1)	910 (94.0)	803 (94.3)	771 (90.5)	< 0.001
Yes	283 (4.8)	95 (2.9)	58 (6.0)	49 (5.8)	81 (9.5)	
**Number of chronic diseases**						
No diseases	523 (8.8)	384 (11.7)	77 (7.9)	42 (4.9)	20 (2.4)	< 0.001
1–3 diseases	3,918 (65.9)	2,230 (70.8)	627 (64.6)	513 (60.2)	458 (53.8)	
≥4 diseases	1,509 (25.4)	572 (17.5)	266 (27.4)	297 (34.9)	374 (43.9)	
SPPB Score (0–12)	6.25 ± 3.36	7.13 ± 3.12	6.25 ± 3.38	5.07 ± 3.06	3.85 ± 3.03	< 0.001

FOF, fear of falling; SPPB, Short Physical Performance Battery.

###  3.2. Falls and FOF independently and combinedly predicted functional limitations

**Figure 1:**
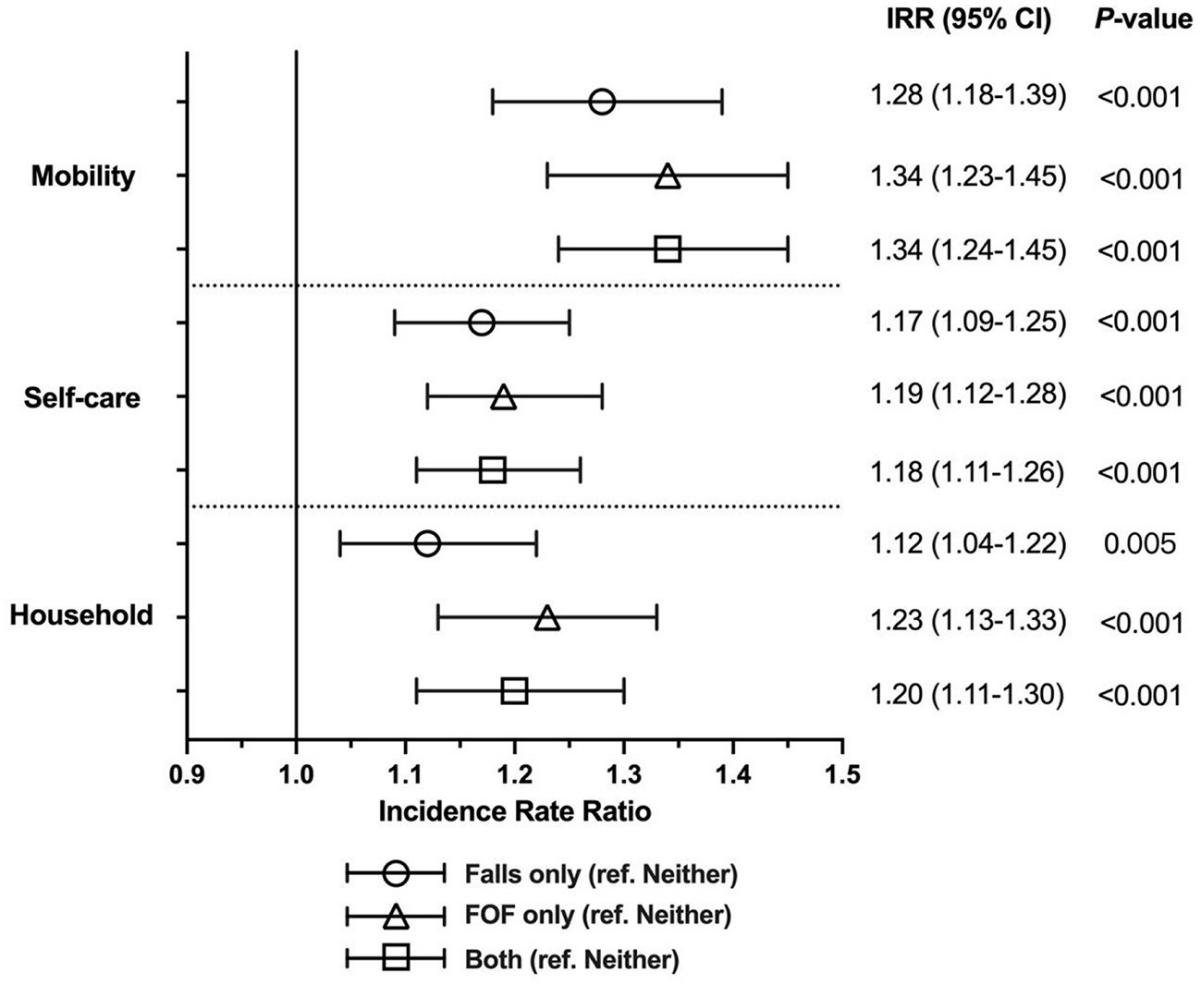
Forest plot depicting fully adjusted Poisson regression analysis of baseline falls and FOF status on functional limitations at R2. Models adjusted for sociodemographic factors (age, sex, race/ethnicity, education) and health-related factors (obesity, depressive symptoms, anxiety symptoms, bothersome pain, visual impairment, dementia, hospitalization, number of chronic diseases, and Short Physical Performance Battery) and outcome of interest at baseline. FOF, fear of falling; IRR, incidence rate ratio; CI, confidence interval.

Figure 1 depicted the longitudinal association of functional limitations with falls and FOF after adjusting baseline sociodemographic and health-related covariates and the outcomes of interest. Compared to neither, both, falls only and FOF only had increased risks of mobility activities limitations, self-care activities limitations and household activities limitations.

### 3.3. Living alone moderated the longitudinal associations of falls and FOF with functional limitations

Table 2 presented the results of the three adjusted Poisson regressions, which examined whether living alone moderated the longitudinal relationship between combined falls and FOF and functional limitations. Living alone moderated the longitudinal associations of falls and FOF with mobility limitations (*P*_interaction_ < 0.01). In contrast, no moderation effect was observed in self-care and household activities limitations, indicating that living alone did not moderate the longitudinal associations of falls and FOF with self-care and household activities limitations.

**Table 2:** Fully adjusted Poisson regression examining the association of living alone and concurrent falls and FOF at baseline with functional limitations outcomes at follow-up.

	**Mobility** ^**a**^		**Self-care** ^**b**^		**Household** ^**c**^	
	**IRR (95% CI)**	***P*-value**	**IRR (95% CI)**	***P*-value**	**IRR (95% CI)**	***P*-value**
**Falls and FOF**						
Neither	1.00 [Ref]	NA	1.00 [Ref]	NA	1.00 [Ref]	NA
Falls only	1.38 (1.24–1.52)	**< 0.001**	1.21 (1.11–1.32)	**< 0.001**	1.23 (1.11–1.38)	**< 0.001**
FOF only	1.36 (1.23–1.50)	**< 0.001**	1.21 (1.11–1.31)	**< 0.001**	1.38 (1.23–1.54)	**< 0.001**
Both	1.43 (1.31–1.57)	**< 0.001**	1.18 (1.08–1.28)	**< 0.001**	1.40 (1.26–1.56)	**< 0.001**
**Living alone**						
No	1.00 [Ref]	NA	1.00 [Ref]	NA	1.00 [Ref]	NA
Yes	1.10 (1.00–1.22)	0.056	1.01 (0.93–1.09)	0.898	1.10 (0.98–1.23)	0.102
**Falls and FOF** × **Living alone**						
Neither × Living alone	1.00 [Ref]	NA	1.00 [Ref]	NA	1.00 [Ref]	NA
Falls only × Living alone	0.82 (0.70–0.96)	**0.013**	0.89 (0.77–1.02)	0.092	0.92 (0.78–1.09)	0.346
FOF only × Living alone	0.96 (0.82–1.11)	0.199	0.96 (0.84–1.10)	0.561	0.94 (0.81–1.10)	0.443
Both × Living alone	0.84 (0.73–0.96)	**0.010**	1.01 (0.90–1.14)	0.843	0.92 (0.79–1.06)	0.242

FOF, fear of falling; IRR, incidence rate ratio; CI, confidence interval.

a Adjusted for sociodemographic factors (age, sex, race/ethnicity, education), health-related factors (obesity, depressive symptoms, anxiety symptoms, bothersome pain, visual impairment, dementia, hospitalization, number of chronic diseases, and Short Physical Performance Battery) and mobility activity limitation level at baseline.

b Adjusted for sociodemographic factors, health-related factors, and self-care activity limitation level at baseline.

c Adjusted for sociodemographic factors, health-related factors, and household activities limitation level at baseline. Bold values means that the number is statistically significant.

**Figure 2:**
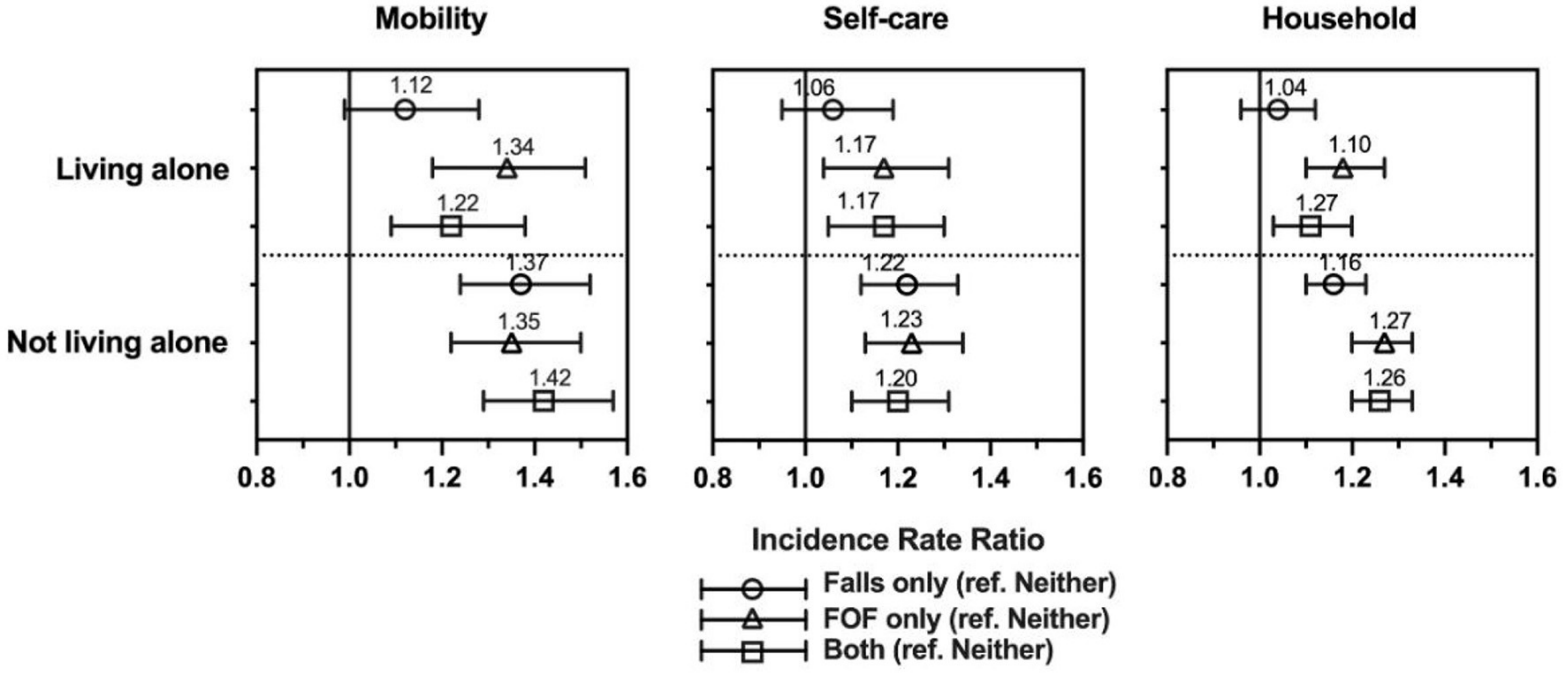
Association of baseline falls and FOF status and follow-up functional limitations stratified by living arrangement. Models adjusted for sociodemographic factors (age, sex, race/ethnicity, education) and health-related factors (obesity, depressive symptoms, anxiety symptoms, bothersome pain, visual impairment, dementia, hospitalization, number of chronic diseases, and Short Physical Performance Battery) and outcome of interest at baseline. FOF, fear of falling; IRR, incidence rate ratio.

Based on the stratified analysis of living alone (Figure 2), compared to neither, falls only did not statistically significantly predict mobility (IRR = 1.12, 95% CI = 0.99–1.28, *P* = 0.08), self-care (IRR = 1.06, 95% CI = 0.95–1.19, *P* = 0.30), and household activities (IRR = 1.04, 95% CI = 0.96–1.13, *P* = 0.31) limitations in older adults who lived alone. However, among those who did not live alone, falls only was associated with a higher risk of functional limitations, with an IRR of 1.37 for mobility (95% CI = 1.24–1.52), 1.22 for self-care (95% CI = 1.12-1.33), and 1.16 for household (95% CI = 1.10–1.23) (all *P* < 0.05). Among those who lived alone or not, both and FOF only were at a higher risk of mobility, self-care and household activities limitations than neither (all *P* < 0.05).

## 4. Discussion

To our knowledge, this longitudinal study is the first one to simultaneously investigate the independent and combined effect of falls and FOF on functional limitations (including mobility, self-care, and household activities limitations) as well as whether living alone moderated these effects using a nationally representative sample of community-dwelling older adults in the US. The findings suggested that falls and FOF independently and combinedly predicted functional limitations and that living alone moderated the longitudinal associations of falls and FOF with mobility activities limitations. Our findings highlighted that we should identify older adults with falls or FOF who do not live alone and develop targeted interventions to prevent functional limitations.

Our study confirmed previous findings that falls and FOF independently predicted functional limitations and further demonstrated the combined effect of the two on functional limitations. Previous studies have only investigated the independent predictive roles of falls and FOF (10, 13, 36–38) and found a strong independent association between falls and functional limitations in older adults, especially for those who experience multiple falls and fall injuries (13, 36, 38). FOF is also an important risk factor for functional limitations (37). Two longitudinal studies found that older adults with FOF had significantly reduced functions (10, 39). A long duration of FOF was associated with a higher risk of decreased activities of daily living (ADL) (10, 40). Older adults with FOF are cautious in performing activities, thereby further reducing their active time (12). Previous studies demonstrated that self-limiting behaviors led to physical deterioration and increased the risk of functional limitations (12, 41), which could explain why FOF causes functional limitations. Furthermore, with the complex causal relationship between falls and FOF (14) the focus of our study was to examine the combined effect of falls and FOF on functional limitations. Individuals who have fallen may subsequently develop FOF, which has been shown to be a direct consequence of falls. Individuals who fell might also experience previous FOF, suggesting it was a risk factor for falls (16). It is reasonable that poor fitness levels resulting from persistent FOF not only develop functional limitations but also increase the likelihood of future falls, which may reinforce the association of FOF with functional limitations. Similarly, in older adults with a history of falls, FOF also strengthened the predictive role of falls on functional limitations (14, 23). Therefore, it is not surprising that in our study, older adults with concurrent falls and FOF are at higher risks of functional limitations compared to neither.

We found that living alone moderated the association of combined effect of falls and FOF with mobility activities limitations. Among the two groups of older adults in our study (fall only and both), those who lived with others have a higher risk of mobility limitation than those who lived alone. The results of falls only showed that the risk of mobility limitations was not significant in older adults who lived alone. To date, only few studies have investigated the relationship between living alone, falls, FOF, and mobility (42, 43). In general, living alone has an impact on the frequency of falls and the occurrence of FOF in older adults (20, 44). One possible explanation is that older adults who live alone are more likely to receive less social support and thus are more likely to feel lonely and isolated, thereby increasing their risk of falls and FOF (45, 46). However, not all older adults who live alone experience loneliness or social isolation. Living alone has been demonstrated to provide some protection against functional limitations in older adults (47). A longitudinal study found that older adults living with others had more limited mobility than those who lived alone (48) because living alone to some extent forces older adults to learn to maintain a high degree of independence and self-management, a phenomenon called “biologically conditioned reflex” (49). If older adults have someone else to rely on, they may give up some opportunities of performing independent activities more easily, resulting in increased functional limitations (50). In this study, living alone reduced the risk of functional limitations in older adults with falls and FOF. Moreover, the choice to live alone could be explained by economic and cultural factors (24). Older adults with greater cultural fit and financial resources are more likely to live alone and have more independence and confidence, which may help them overcome mobility restrictions due to falls and/or FOF.

This study has important implications for research, practice, and policy on the prevention and management of functional limitations in older adults. Recognizing the combined effect of previous falls and FOF on functional limitations, clinicians should regularly examine patients with both falls and FOF on their risk of developing functional limitations. Additionally, the moderating role of living alone found in the study calls for particular attention to developing functional limitations prevention interventions for older adults with falls and FOF tailored to their living arrangement (living alone or not). This has important implications for policymakers, clinicians, and family members.

Several limitations of this study should be noted. First, the reliability and validity of measuring FOF by asking participants if they were worried about falling in the last month remain unknown. Second, measures of falls are through retrospective self-report and may suffer from recall bias and reporting errors. For older adults, the one-year fall recall window may be too long. They may only remember their injured falls. Third, the covariates we identified were limited to those collected from the NHATS database, and thus residual confounding may exist. Fourth, our study could not provide causal inference despite with longitudinal study design. However, the study has undeniable strengths. We used nationally representative longitudinal data to examine the temporal impact of falls and FOF on functional limitations. We also innovatively explored the moderating effects of living alone (yes/no) and adjusted a comprehensive list of covariates.

## 5. Conclusions

Our study found the independent and combined effect of falls and FOF on functional limitations and the moderating role of living alone. While making efforts to prevent falls and FOF in older adults, the government, clinicians, and caregivers should consider the social background to help older adults prevent and manage functional limitations.

## Data availability statement

The original contributions presented in the study are included in the article/supplementary material, further inquiries can be directed to the corresponding author.

## Ethics statement

The studies involving human participants were reviewed and approved by the Johns Hopkins University Institutional Review Board. The patients/participants provided their written informed consent to participate in this study.

## Author contributions

KL: writing—original draft and writing—review and editing. WP: conceptualization, methodology, data analysis, and writing—original draft. SG, CL, YZ, and XH: writing—review and editing. ML: methodology and writing—review and editing. All authors contributed to the article and approved the submitted version.
